# Assessment of the complaints and the hospital application of patients with acute coronary syndrome, and the patient's knowledge and behaviors regarding the management of cardiovascular risk factors

**DOI:** 10.4314/ahs.v25i1.19

**Published:** 2025-03

**Authors:** Busra Zehra Buyukkilic, Semiha Akin Eroglu

**Affiliations:** University of Health Sciences, Hamidiye Faculty of Nursing, Department of Internal Medicine Nursing

**Keywords:** Acute coronary syndrome, hospital admission, cardiovascular risk factors, knowledge, behaviors

## Abstract

**Background:**

This research was carried out to determine the complaints and duration of hospital admission of patients with acute coronary syndrome (ACS) and to evaluate their healthy lifestyle behaviors related to the management of cardiovascular risk factors.

**Methodology:**

This is a descriptive cross-sectional study. The study sample consisted of 202 patients diagnosed with ACS from September 2022 to August 2023. Data were collected using the Hospital Admission and Symptom Assessment Survey, Cardiovascular Disease Risk Factor Diagnostic Survey, Healthy Lifestyle Behaviors Scale II (HPLP II), and Cardiovascular Disease Risk Factors-Knowledge Level (CARRF-KL) scale.

**Results:**

The median duration of decision-making for admission to the hospital was 40 minutes, and the mean duration until arrival at the hospital was 17.35 ± 9.76 minutes. There was no statistically significant difference between the duration of admission decisions according to the association of cardiac complaints with heart disease (p > 0.05). The CARRF-KL total scale mean score was calculated to be 19.98 ± 4.41, and the HPLP II total scale mean score was 107.69 ± 18.09. A positive and statistically significant correlation was found between CARRF-KL total scale and HPLP II total scale scores (r = 0.49, p < 0.05).

**Conclusion:**

Although the duration of admission to the hospital for individuals diagnosed with ACS was within the period recommended by the guidelines, it is noteworthy that they did not associate their complaints with heart defects in their decisions to be admitted to the hospital.

## Introduction

According to the World Health Organization, coronary artery disease (CAD) is the leading cause of death worldwide. It is responsible for three-quarters of all deaths in low- and middle-income countries, killing approximately 17.8 million individuals each year^1^. CAD is estimated to be present in 1.845 out of every 100,000 people by 2030 and is reported to cause 9 million deaths around the world^2^. As stated in the data from the Turkish Statistical Institute, 35.4% of all deaths occurring in 2022 were reported to be due to circulatory system diseases, and a large proportion of this rate was reported to originate from ischemic heart diseases^3^.

Given the fact that it has a high incidence among cardiovascular diseases and is the leading cause of mortality in Turkey, patients diagnosed with acute coronary syndrome (ACS) should be evaluated physically, socially, and psychologically in a multidimensional manner^4^. In a study conducted by Dogan et al. in 2022, it was documented that the success of treatment in ACS was closely related to factors such as early admission of the patient to the hospital, level of knowledge about cardiovascular risk factors, and healthy lifestyle^5^.

The length of time between symptom onset and initiation of treatment is the sum of the time between an individual's decision to seek medical help and the time it takes to reach the hospital. Delays until the first medical intervention is provided are defined as patient-caused delays, while delays from first medical contact to diagnosis and from diagnosis to the start of reperfusion treatment are considered system-caused delays^6^. In research carried out by Arrebola-Moreno and his colleagues in 2020, it was found that the most important determinant in the mortality rates and recovery process associated with myocardial infarction (MI) was “the delays in decision-making” and that the time lost in decision-making is mainly constituted in the process of deciding whether to admit to a hospital^7^.

The duration of the decision-making process to seek medical help varies according to many factors, such as age, gender, and economic status. Particularly in women, the elderly, those with a history of diabetes, and those with low socioeconomic status, the duration of hospital admission is reported to be much longer^8^. It is suggested that personality traits, the typicality and severity of symptoms, the patient's recognition of cardiac findings, and the belief of the patient that his/her disease is treatable are effective in reducing the duration of admission^9^. Sociocultural structure, perception of health, coping methods, and level of knowledge about cardiovascular risk factors have also been shown to be effective in the duration of hospital admission^10^.

The level of knowledge about risk factors for cardiovascular diseases and healthy lifestyle habits regarding the management of these risk factors are thought to be effective in patients' faster decision-making to seek medical help. To reduce mortality, raise public awareness of CVD risk factors, and prevent delays in hospital admissions, increasing the level of knowledge of individuals about the symptoms of ACS should be among the priorities of healthcare professionals.

Although the effect of healthy lifestyle habits on the duration of hospital admission is not fully known, it has been pointed out that these habits play a protective role in the prevention of cardiovascular diseases and in reducing the risk of disease. Accordingly, this study was implemented to determine the complaints and duration of hospital admission and to evaluate healthy lifestyle habits regarding the management of cardiovascular risk factors in patients treated for ACS.

The findings obtained from this research will provide important data about individuals diagnosed with ACS when it comes to identifying their complaints and the duration of their admission and to determining the factors affecting the duration of admission. It is anticipated that the results of this study will increase the knowledge level of individuals diagnosed with ACS regarding the management of risk factors, prevention of delays in hospital admission, and development of healthy lifestyle habits.

These research findings may guide health professionals in the development of approaches to reduce hospital admission delays in individuals with cardiovascular disease and in the development of multidisciplinary projects on the management of patient's cardiovascular risk factors. Furthermore, it is thought that these results will contribute to raising awareness to ensure faster access of individuals to the hospital, raising awareness of the 112-emergency team and healthcare professionals about the importance of early admission in ACS cases, and raising awareness in society about CVD risk factors and symptoms of ACS.

This research was carried out to determine the complaints and duration of hospital admission of patients with ACS and to evaluate their healthy lifestyle behaviors related to the management of cardiovascular risk factors. The findings obtained from this research will contribute to the scope of determination of the complaints, the duration, and the factors affecting the admission process while allowing us a better understanding of the importance of early hospital admission.

### Research Questions

1. What is the duration from symptom onset to hospital admission in patients diagnosed with ACS?
2. What are the hospital admission complaints of patients diagnosed with ACS?
3. What is the level of knowledge of patients with ACS about cardiovascular risk factors?
4. What are the healthy lifestyle habits of patients diagnosed with ACS?
5. Is there a difference between the Healthy Lifestyle Behavior Scale II (HPLP II) scores of patients diagnosed with ACS in terms of hospital admission characteristics and complaints?
6. Is there any correlation between healthy lifestyle behaviors and knowledge of patients with ACS about cardiovascular disease risk factors?

## Methodology

### Design and setting

This research is a descriptive cross-sectional study. The research was carried out by the cardiology service of a training and research hospital in Istanbul.

### Population and sample

The research population consisted of individuals diagnosed with ACS who were hospitalized in the cardiology service of a training and research hospital in Istanbul within one year. The research sample consisted of Turkish-speaking patients who volunteered to participate in the study and who underwent percutaneous coronary intervention with a diagnosis of ACS in the cardiology service between September 2022 and August 2023. Patients were selected using the convenience sampling method. A total of 246 patients were contacted in the study. Among these 246 patients, 30 patients were excluded from the study group due to not meeting the inclusion criteria and 14 patients were not included in the research owing to lack of voluntary consent to take part in the study. Therefore, a total of 202 ACS patients between September 2022 and August 2023 constituted the study sample.

### Data collection

The data were collected by the researcher through face-to-face interviews in a room with patients diagnosed with ACS. Datasheets took approximately 25–30 minutes to fill out. The data were obtained using four data collection tools. Patient Information Survey: This consists of 12 questions to identify sociodemographic features, healthy living habits, disease, and treatment-related characteristics.

Hospital Admission and Symptom Assessment Survey: In this data collection tool, the complaints and severity of individuals diagnosed with ACS before admission to the hospital, the time and location of the onset of complaints, the duration and method of transportation to the hospital, individual perceptions of the hospital admission process, and interventions to cope with cardiac complaints are questioned.

Cardiovascular Disease Risk Factors Assessment Survey: This survey contains 13 questions. Through this form, the history of the individual's first-degree relatives and himself/herself, history of cardiac risk factors (diabetes, hypertension, and hypercholesterolemia) are questioned. Questions are answered as “Yes” or “No.” This questionnaire contained questions about the individual's smoking and alcoholic beverage habits, physical activity habits, perception of stress level, amount of salt intake in their meals, and body mass index.

Cardiovascular Disease Risk Factors-Knowledge Level (CARRF-KL) Scale: This scale is the first of its kind in Turkey and can be used to determine the level of knowledge of individuals about CVD risk factors. Of the scale, the first four items are related to the characteristics of CVDs, the preventability of risk factors, and the age factor, while 15 items (items 5, 6, 9–12, 14, 18–20, 23–25, 27, and 28) question the risk factors, and nine items (items 7, 8, 13, 15, 16, 17, 21, 22, and 26) question the impact of change in risk behaviors.

The scale items are in the form of a complete sentence that can be either true or false. Questions are answered as “Yes” and “No.” Six of the statements (items 11, 12, 16, 17, 24, and 26) in the scale are negative items. Each correct answer has a point value of 1. The lowest score that can be attained from the scale is zero, and the highest total score is 28. A higher score on the scale indicates that individuals have a higher level of knowledge about cardiovascular disease risk factors^11^. The scale was developed by Arikan and colleagues in 2009. Cronbach's alpha internal consistency coefficient was 0.77. In this study, the Cronbach's alpha internal consistency coefficient was 0.83.

Healthy Lifestyle Behavior Scale II: The first version of the scale developed by Walker in 1987 was a 4-point Likert scale consisting of 48 items and six factors. The scale was renewed in 1996 and named the Healthy Lifestyle Behaviors Scale II (HPLP II). (12) The scale consists of six subdimensions (responsibility for health, physical activity, nutrition, spiritual development, interpersonal relationships, and stress management). All scale items are positive. Scale items are graded as “(1) never,” “(2) sometimes,” “(3) often,” and “(4) regularly.” The lowest score that can be attained from the scale is 52, and the highest score is 208. The lowest and highest scores for the subdimensions are as follows: 9–36 for the responsibility of health subdimension, 8–32 for the physical activity subdimension, 9–36 for the nutrition subdimension, 9–36 for the spiritual development subdimension, 9–36 for the interpersonal relationships subdimension, and 8–32 for the stress management subdimension. It is stated that the higher the score attained from the scale, the healthier the lifestyle habits individuals have. In a validity and reliability study of the scale in Turkish (Bahar et al., 2008), the internal consistency reliability coefficient of the scale was found to be 0.92. (12) In this study, the internal consistency coefficient of the scale was 0.95.

### Data Evaluation and Statistical Analysis

Statistical analysis of the data was performed using the IBM SPSS for Windows version 21.0 package program. For the statistical analyses, the application R. Vers. 2.15.3 (R Core Team, 2013) was utilized. The study data were analyzed using minimum, maximum, mean, standard deviation, median, first quarter, third quarter, frequency, and percentage. The conformity of quantitative data to normal distribution was evaluated by the Shapiro–Wilk test and graphical analysis.

As the independent group t-test was utilized for evaluations between two groups, the one-way analysis of variance and Dunn-Bonferroni test were utilized for evaluations between more than two groups for variables with normal distribution. For variables that did not display normal distribution, the Mann–Whitney U test was utilized for evaluations between two groups, whereas the Kruskal–Wallis test and the Dunn–Bonferroni test were utilized for evaluations between more than two groups. The Pearson correlation coefficient was utilized to determine the level of correlation between the quantitative data. Statistical significance was taken as p < 0.05.

### Ethical aspects of the research

Approval was granted by the Ethics Committee for the implementation of the study (Decision Date: 17/06/2022, number: 16/14). Institutional authorization was received (Decision Date: 12/09/2022, number: 161768). Permission to use the scale was granted before the study began.

With the informed voluntary consent form, the approval of the patients was obtained. The World Medical Association Declaration of Helsinki and Good Clinical Practice guidelines were faithfully respected throughout the research.

## Results

### Personal and disease-related characteristics

The mean age of the examined patients was 64.63 ± 11.27 years. Of the sample, 64.9% were male, of which 36.2% were literate or graduated from primary school. Among the inspected patients, 16.3% had MI, 34.7% had diabetes, 55% had hypertension (HT), and 30.7% had hyperlipidemia.

Of the study cohort, 38.6% were treated with USAP (Unstable Angina Pectoris), 47% with non-ST-elevation myocardial infarction (NSTEMI), and 14.4% with ST-elevation myocardial infarction (STEMI) STEMI (Table 1).

**Table 1:** Demographic Characteristics of Patients Treated for Acute Coronary Syndrome (N = 202)

Variables	Ort ± sd	Min-Max (Median)
**Age (year)**	64.63 ± 11.27	36-91 (66)

	**n**	**%**

**Sex**		

Female	71	35.1

Male	131	64.9

**Marital status**		

Married	152	75.2

Single	50	24.8

**Level of education**		

Illiterate	27	13.4

Literate or primary school graduate	73	36.2

Secondary school graduate	51	25.2

High school graduate	36	17.8

University graduate and above	15	7.4

**Employment status**		

Working	45	22.3

Not working	157	77.7

**Income status**		

Income less than expenditure	50	24.7

Income matches expenditure	148	73.3

Income more than expenditure	4	2.0

**Perceptions of daily stress situation**		

“I am not stressed”	1	0.5

“Slightly”	38	18.8

“Moderate”	107	53.0

“Very”	38	18.8

“Too much”	18	8.9

**Body Mass Index**		

Normal (18.5-24.99 kg/m^2^)	42	20.8
Overweight (25.0-29.99 kg/m^2^)	97	48.0
Obese (30.0-34.99 kg/m^2^)	61	30.2
Morbidly obese (40.0-above kg/m^2^)	2	1.0

**Regular attendance to health checkups**		

No	138	68.3
Yes	64	31.7

**Frequency of regular health check-ups (n = 64)**		

Once a month	3	4.7
Every three months / Quarterly	19	29.7
Once a year	22	34.4
Other (Based on my complaint and physician's recommendation)	20	31.2

	**Mean ± sd**	**Min-Max (Median)**

Duration (in years) of tobacco use (n = 115)	31.04 ± 12.39	2-60 (30)

Amount of tobacco use (days/piece) (n = 115)	22.14 ± 13.41	7-80 (20)

Duration (in-years) of alcohol use (n = 46)	3.33 ± 2.52	0.5-12 (2.5)

Body Mass Index (kg/cm^2^)	28.34 ± 3.99	20.24-40.32 (27.84)

Date of first diagnosis of Acute Coronary Syndrome (months) (n = 113)	122.52 ± 99.83	3-384 (96)

Date of Coronary Angiography application (month)	98.8 ± 87.87	3-384 (84)

Date of Percutaneous Coronary Angioplasty-Stenting (month)	87.42 ± 67.18	6-240 (72)

Date of Coronary Artery Bypass Graft surgery (month)	112.4 ± 97.83	12-324 (60)

### Duration from symptom onset to hospital admission

The mean time for patients to decide on admission to the hospital was 648.84 ± 1615.02 minutes. The average time until arrival at the hospital was calculated to be 17.35 ± 9.76 minutes. Of the total, 30.7% of the study cohort thought that their admission to the hospital was late. In the cohort, 77.4% reported that they did ignore the complaints, and 19.4% reported that they experienced delays in arriving at the hospital due to the distance between their home and the hospital.

### Hospital admission complaints

The complaints of the patients admitted to the hospital are shown in Table 2. Of the study cohort, 37.6% (N = 76) reported that they were in a state of rest at the onset of symptoms, 5% (N = 10) were practicing sports, and 26.7% (N = 54) were doing housework. Of the total, 55% (N = 111) of the patients indicated that before admission to the hospital, they had taken their interventions. Within this cohort, 18.9% reported that they took their medication, 36.9% reported that they massaged their chest and arms, 62.2% reported that they rested/stretched, and 18% reported that they called the ambulance. Only 63.4% of the patients reported that they associated their symptoms with heart disease (Table 3).

**Table 2:** Complaints and Duration of Hospital Admission In Patients Treated for Acute Coronary Syndrome (N = 202)

	n	Duration of hospital admission decision-making Median (Q1, Q3)	^b^Test value (*χ*^2^)	p
**Excessive perspiration**			32.720	<0.001*
None	93	40 (20, 180)		
Slight	13	60 (20, 360)		
Moderate	48	120 (35,390)		
Severe	48	12.5 (5, 30)		
**Feverishness**			23.318	<0.001*
None	102	40 (20, 180)		
Slight	16	150 (20,360)		
Moderate	49	120 (30, 420)		
Sever	35	10 (5, 30)		
**Fainting**			21.660	<0.001*
None	171	60 (20, 300)		
Slight	13	20 (10, 40)		
Moderate	7	10 (5, 50)		
Severe	11	5 (0, 10)		
**Nausea and vomiting**			14.871	0.002*
None	141	60 (20, 360)		
Slight	28	42.5 (20, 90)		
Moderate	25	20 (10, 120)		
Severe	8	7.5 (2.5, 15)		
**Chest pain**			11.720	0.008*
None	28	40 (20, 150)		
Slight	3	30 (10, 240)		
Moderate	64	60 (30, 360)		
Severe	107	30 (10, 180)		
**Powerlessness**			13.221	0.004*
None	76	42.5 (20, 360)		
Slight	12	360 (90, 2160)		
Moderate	74	37.5 (20, 120)		
Severe	40	30 (7.5, 120)		
**Dyspepsia**			1.220	0.75
None	116	37.5 (15, 180)		
Slight	38	60 (20, 360)		
Moderate	40	35 (17.5, 450)		
Severe	8	90 (45, 120)		
**Restlessness-temperance**			14.263	0.003*
None	61	40 (20, 240)		
Slight	12	300 (75, 510)		
Moderate	79	45 (20, 240)		
Severe	50	25 (5, 120)		
**Heart palpitations**			0.824	0.84
None	82	60 (15, 240)		
Slight	23	30 (20, 120)		
Moderate	53	30 (15, 300)		
Severe	44	40 (12.5, 180)		
**Shortness of breath**			1.674	0.64
None	90	42.5 (20, 180)		
Slight	13	40 (10, 50)		
Moderate	49	40 (20, 240)		
Severe	50	30 (15, 600)		
**Cold Sweats**			13.565	0.004*
None	82	60 (30, 360)		
Slight	9	30 (10, 30)		
Moderate	57	60 (20, 180)		
Sever	54	20 (10, 120)		

**Table 3:** Coping Behaviours of Patients Treated for Acute Coronary Syndrome with Their Complaints Leading to Hospital Admission (N = 202)

	n	%
**Self-intervention status of the individual before admission to the hospital**		
No	91	45.0
Yes	111	55.0
**Type of self-intervention performed by the individual before hospital admissiont**		
“I laid down and rested.”	69	62.2
“I took a break from what I was doing.”	48	43.2
“I massaged my chest and my arm.”	41	36.9
“I measured my blood pressure”	41	36.9
“I called a relative”	27	24.3
“I took my medicine”	21	18.9
“I called an ambulance.”	20	18.0
“I set off for the hospital.”	7	6.3
“I drank water ”	6	5.4
“I take an appointment from a family practitioner”	5	4.5
“I went back to my house.”	3	2.7
“I applied heat to the area of pain.”	2	1.8
“I called my doctor.”	2	1.8
Other (coughing, applying muscle relaxants)	2	1,8
**Mode of transportation to the hospital**		
Private vehicle	107	53.0
Ambulance	67	33.2
Public transportation	17	8.3
“I went by foot”	10	5.0
“I was in the hospital when the symptoms began.”	1	0.5
**Individual perception of delay in hospital admission**		
“I didn't delay in admitting to the hospital”	140	69.3
“I was late in admitting to the hospital”	62	30.7
**Reason for delay in hospital admission†**		
“I ignored the complaints”	48	77.4
“I didn't think it was a heart problem”	36	58.1
“My home is far away”	12	19.4
“I thought it would resolve after I took my medicine”	11	17.7
“Due to heavy traffic”	9	14.5
“I couldn't find anyone to help me”	3	4.8
“The ambulance didn't come”	1	1.6
Other (I couldn't find an appointment; I thought my complaint would be resolved)	6	9.7
**Associations of complaints with heart disease**		
Yes, I've made the connection.	128	63.4
No, I didn't associate it.	74	36.6
	**Ort ± ss**	**Min-Maks (Medyan)**
Decision time for hospital admission (in min)	648.84 ± 1615.02	0-10080 (40)
Length of time until arrival at the hospital (min)	17.35 ± 9.76	0-60 (15)
Duration of Percutaneous Coronary Intervention (min)	1415.36 ± 1261.49	5-5760 (1080)

There was no statistically significant correlation between the percentage of associating complaints with heart disease and the duration of hospitalization decisions according to educational level (p > 0.05). It was found that the duration of hospital admission decisions for those with severe chest pain and complaints was shorter than the duration of hospital admission decisions for those with moderate chest pain (p = 0.004) (Table 3).

The prevalence of patients who used medication for their complaints before applying to the hospital was found to be 10.39%. The proportion of those who reported using analgesics at the onset of their complaints was 3.9%, and the rate of those using anticoagulants or antiaggregants was 0.4%.

### Knowledge of patients with ACS about cardiovascular disease risk factors and associated variables

The research sample had an average score of 2.28 ± 0.95 on the characteristics of the Cardiovascular Disease Risk Factors-Knowledge Level (CARRF-KL) CVD's subscale, 11.55 ± 2.66 on the risk factors subscales, and 6.14 ± 1.65 on the outcome of change in risk behaviors subscales. The total mean CARRF-KL score was 19.98 ± 4.41. The CARRF-KL risk factors and the outcome of changes in risk behaviors subscale scores of male patients were reported to be higher than those of female patients. The scores of illiterates on the subscales of risk factors and outcome of change in risk behaviors and total scale were identified to be lower than the scores of literate and primary school graduates, secondary school graduates, high school graduates, and those with university and higher education (p < 0.05).

There was no statistically significant correlation between the duration of time until the decision for admission to the hospital and the subscales and total scale scores of the CARRF-KL scale (p > 0.05). No statistically significant correlation was found between the duration of time until arrival at the hospital and the CARRF-KL subscale and total scale scores (p > 0.05) (Table 4).

**Table 4:** Comparison of CARRIF-KL and HPLP II Scores Based on The Duration of Hospital Admission (N = 202)

	Variables	Duration of hospital admission decision-making (min)		Length of time until arrival at the hospital (min)	
**CARRIF-KL**		r	p	r	p
	Characteristics of cardiovascular diseases	0.08	0.24	0.10	0.14
	Risk factors	0.07	0.28	0.01	0.82
	The Outcome of a change in Risk behaviors	0.13	0.05	0.11	0.12
	Total Scale	0.11	0.11	0.07	0.30

**HPLP II**	Responsibility for health	0.003	0.97	-0.159	0.024*
	Physical Activity	-0.027	0.70	-0.059	0.41
	Nutrition	0.073	0.30	0.075	0.29
	Spiritual Development	0.074	0.29	0.028	0.70
	Interpersonal relationships	0.067	0.34	0.023	0.75
	Stress Management	0.045	0.53	0.067	0.35
	Total Scale	0.044	0.53	-0.012	0.87

The characteristics of the CVD subscale and total scale scores of those who used medication before admission to the hospital were found to be higher than those of the patient group who applied other interventions when the patients' complaints started (p < 0.05). The scores of the characteristics and subscale of CVDs of those who were admitted to the hospital with a delay as a result of thinking that their complaints would resolve due to taking their medication were found to be higher than the scores of those who thought that they were admitted to the hospital for other reasons (p < 0.05). No statistically significant correlation was found between the total scale scores based on the association of complaints with heart disease (p > 0.05) (Table 4).

### Healthy lifestyle behaviors of patients with ACS

The mean score value of the HPLP II responsibility for health subscale was identified as 15.21 ± 3.68 points, the mean score value of the physical activity subscale was determined as 11.29 ± 4.60 points, the mean score value of the nutrition subscale was calculated as 21.26 ± 3.11 points, and the mean score value of the spiritual development sub-scale was 21.76 ± 3.84 points. The mean score in the HPLP II interpersonal relations subscale was determined as 21.24 ± 3.89, the mean score in the stress management subscale was 16.92 ± 3.20, and the mean score in the total scale was 107.69 ± 18.09.

A negative statistically significant correlation was found between age and HPLP II responsibility for healthy physical activity, nutrition, spiritual development, interpersonal relationships, stress management subscale, and total scale scores (p < 0.05). The HPLP II responsibility for health subscale scores of male patients were found to be higher than those of female patients (p < 0.05). The HPLP II responsibility for health subscale scores of illiterates were found to be lower than those of middle school and high school graduates and those with university and higher education (p < 0.05). Physical activity subscale scores and HPLP II total scale scores of illiterates were found to be lower than those of literates, primary school graduates, secondary school graduates, high school graduates, and those with university and higher education (p < 0.05).

Responsibility for health and physical activity subscale scores of those diagnosed with STEMI were found to be higher than those of those diagnosed with USAP and NSTEMI (p < 0.05). Stress management subscale scores of patients diagnosed with NSTEMI were found to be lower than those of USAP and STEMI patients (p < 0.05). A statistically significant negative correlation was detected between the duration of ACS diagnosis and HPLP II responsibility for health, physical activity, nutrition, spiritual development, interpersonal relationships, stress management sub-scale, and total scale scores (p < 0.05). admission characteristics and complaints and eating (p < 0.05) (Table 4). delayed their admission to the hospital were found to be higher than those who did not think that they had delayed their admission (p < 0.05) (Table 4). Scores of those who associated their complaints with heart disease were found to be lower than the scores of those who did not associate their complaints with heart disease (p < 0.05).

### Comparison of Healthy Lifestyle Behaviors Scale II (HPLP II) and Cardiovascular Disease Risk Factors-Knowledge Level (CARRF-KL) scale scores

A positive and statistically significant correlation was found between the CARRF-KL risk factors subscale scores of the group and the HPLP II responsibility for health, physical activity, nutrition, spiritual development, interpersonal relationships, stress management subscale, and total scale scores (r = 0.25–0.51; p < 0.05). A statistically significant positive correlation was found between the CARRF-KL risk behavior outcome of change subscale scores and HPLP II responsibility for health, physical activity, nutrition, spiritual development, interpersonal relationships, stress management subscale, and total scale scores (r = 0.30–0.48; p < 0.05) (Table 5). A positive and statistically significant correlation was found between CARRF-KL total scores and HPLP II total scale scores (p < 0.05) (Table 5).

**Table 5:** Findings Related to the Comparison of CARRIF-KL and HPLP Scores of Patients Treated for Acute Coronary Syndrome (N = 202)

		Cardiovascular Disease Risk Factors Knowledge Scale (CARRIF-KL) dimensions			
		Characteristics of cardiovascular diseases	Risk Factors	The Outcome of a change in Risk behaviors	Total Scale
**HPLP dimensions**					
Responsibility for health	r	0.14	0.39	0.36	0.40
	**p**	0.035*	< 0.001*	< 0.001*	< 0.001*
Physical activity	r	0.04	0.24	0.30	0.27
	**p**	0.54	< 0.001*	< 0.001*	< 0.001*
Nutrition subscale	r	0.16	0.27	0.34	0.32
	**p**	0.021*	< 0.001*	< 0.001*	< 0.001*
Spiritual development	r	0.18	0.51	0.48	0.53
	**p**	0.007*	< 0.001*	< 0.001*	< 0.001*
Interpersonal relationships	r	0.16	0.39	0.41	0.42
	**p**	0.021*	< 0.001*	< 0.001*	< 0.001*
Stress management	r	0.17	0.44	0.47	0.48
	**p**	0.012*	< 0.001*	< 0.001*	< 0.001*
Total Scale	r	0.17	0.46	0.48	0.49
	**p**	0.013*	< 0.001*	< 0.001*	< 0.001*

HPLP II: Health-Promoting Lifestyle Profile II

## Discussion

This research was conducted to determine the complaints and duration of hospital admission and to evaluate the level of knowledge about the management of cardiovascular risk factors and healthy lifestyle behaviors of patients treated for ACS. Apart from chest pain, patients with ACS may experience upper extremity pain, neck and jaw pain, discomfort in the epigastric region/epigastrium, dyspnea, nausea, perspiration, and dizziness. In this research, 92.6% of the sample reported experiencing pain. Considering the similarities with some studies (DeVon et al., 2020; Hammer et al., 2019; Ferry et al., 2019), it can be concluded that the most common complaint is pain felt in various parts of the body with different types and intensities^13^–^15^.

Asgari et al. (2022) found that factors such as anxiety, lack of knowledge about ACS symptoms, worry about disturbing others, and associating complaints with noncardiac causes played a role in the decision-making to apply to the hospital^16^. From the research carried out by Raveena et al. (2023), the primary factor contributing to delayed hospital admissions among patients with a prior history of diabetes and a subsequent diagnosis of MI was found to be the failure to correlate their present symptoms with a cardiac condition^17^. Some researchers have claimed that the recognition of symptoms associated with ACS hinges upon the prerequisite cognitive acceptance of the potentiality of experiencing ACS. The findings obtained from this research are in line with the results of previous studies^18^, ^19^. Individuals may not necessarily recognize that their pain or other complaints are indicative of a severe health concern; instead, they may tend to connect such symptoms to other conditions or seek relief through restorative measures. Rather than going to the hospital, they may tend to wait due to the fear and anxiety that they experience. In this regard, it may be suggested that the complaints and perceptions of patients with ACS symptoms at hospital admission are significant in terms of associating their symptoms with a cardiovascular condition.

Within the scope of this research, the duration encompassing the onset of ACS symptoms and patients' admission to the hospital ranged from 0 to 60 minutes, with a calculated mean duration of 17.35 ± 9.76 minutes. Whereas the median value of the duration to decide to apply to the hospital was 40 minutes, the median value of the duration until arrival at the hospital was 15 minutes. It is well established that the early initiation of treatment in cases of ACS is far better for the prognosis of the patient. Contacting the emergency department within 30 minutes of the onset of ACS symptoms allows the patient to be rapidly assessed and appropriate treatment initiated.

In a study carried out by Beza et al. in 2021, it was determined that the average time from symptom onset to the first medical contact was 12.7 hours in ACS patients, and this time ranged between 10 minutes and 96 hours^20^. In a retrospective study conducted on 1,076 STEMI patients in Indonesia, the mean duration from symptom onset to treatment was calculated as 180 minutes, and it was reported that only 6.4% of patients were admitted to the hospital within 60 minutes of symptom onset, with the majority admitted after 180 minutes^21^. After a multicenter study implemented on a cohort consisting of 20.937 ACS patients in India, it was found that the median duration from symptom onset to hospital arrival was approximately six hours. Statistical findings indicated that patients diagnosed with STEMI were admitted to the hospital earlier than NSTEMI or USAP patients^22^. In a study carried out by Dogan in 2022, MI patients were analyzed in two distinct groups: shorter and longer than 30 minutes and shorter and longer than 2 hours. More than half of MI patients were found to be admitted to hospital within 2 hours from the onset of symptoms^23^.

In the context of this research, it was observed that ACS patients under investigation demonstrated noticeably shorter periods for both the decision-making process leading to hospital admission and the actual hospital admission times (min–max 0–60 min) compared to those reported in analogous studies^20^–^22^. The outcome obtained in this research may be associated with the individual characteristics of patients diagnosed with ACS, comorbid conditions and clinical factors, health literacy, transportation conditions and traffic density, financial resources, and emergency services.

In this study, the mean CARRF-KL total scale score of patients diagnosed with ACS was calculated as 19.98 ± 4.41. The mean CARRF-KL scale score in type 2 DM patients was 22.14 ± 2.19 in the study carried out by Bas et al^24^. The mean CARRF-KL scale score in patients with a history of HT and CVD was 20.21 ± 4.39 in the study performed by Ucar et al. (2017)^25^. The subscales of characteristics of CVDs and outcome of change in risk behaviors yielded the lowest scores among this cohort of patients with ACS.

The mean score of the HPLP II total scale was 107.69 ± 18.09. Patients attained the highest scores in the sub-scales of spiritual development, nutrition, interpersonal relationships, stress management, responsibility for health, and physical activity. The mean scores of the participants on the CARRF-KL scale were above the median value; however, the scores on the HPLP II were not at the desired level, indicating that the healthy lifestyle behaviors of individuals diagnosed with ACS participating in the study were not at the desired level.

## Limitations and strengths

Our research draws its strength from a meticulous methodology characterized by direct, face-to-face relationships with patients implemented in a training and research hospital setting. The limitations of the research can be described as follows: first, the study was performed in a singleindividuals diagnosed with ACS.

## Conclusion

Although the duration of admission to the hospital for individuals diagnosed with ACS was within the period recommended by the guidelines, it is noteworthy that they did not associate their complaints with heart defects in their decision to be admitted to the hospital. Although the education level was low in most of the groups, the level of knowledge about cardiovascular risk factors in patients diagnosed with ACS was above the median.
